# Validation and psychometric properties of the Depression Anxiety Stress Scale for Youth in Chinese adolescents

**DOI:** 10.3389/fpsyg.2024.1466426

**Published:** 2024-11-13

**Authors:** Jian Jiang, Jianhua Chen, Zhifeng Lin, Xuwei Tang, Zhijian Hu

**Affiliations:** ^1^Fujian Provincial Hospital, Fuzhou, China; ^2^Department of Epidemiology and Health Statistics, School of Public Health, Fujian Medical University, Fuzhou, China

**Keywords:** DASS-Y, mental health, adolescent, psychometric properties, validation

## Abstract

**Introduction:**

Depression and anxiety are the most common mental health problems among adolescents. The Depression Anxiety Stress Scales for Youth (DASS-Y) is a newly developed instrument designed to assess these problems in adolescents.

**Aim:**

The present study aims to evaluate the psychometric properties of the DASS-Y among Chinese adolescents.

**Methods:**

A total of 326 secondary school students aged 14–18 years participated in the study. A convenience sampling method was adopted to conduct a test–retest of the DASS-Y among Chinese secondary school students. McDonald’s omega, Cronbach’s alpha, and intraclass correlation coefficient (ICC) along with their 95% *CI* were used to assess the internal consistency and test–retest reliability of the DASS-Y. Confirmatory Factor Analysis (CFA) evaluated the structural validity and convergent validity of the DASS-Y through the Comparative Fit Index (CFI), Tucker-Lewis Index (TLI), Root Mean Square Error of Approximation (RMSEA), as well as Average Variance Extracted (AVE) and Composite Reliability (CR). Pearson correlation coefficients with the General Health Questionnaire-12 (GHQ-12), and the Pittsburgh Sleep Quality Index (PSQI) assessed criterion validity.

**Results:**

The CFA confirmed the validity the DASS-Y three-factor model consisting of depression, anxiety, and stress. The internal consistency reliability of the DASS-Y was found to be robust, with McDonald’s omega and Cronbach’s alpha values exceeding 0.8 for all dimensions across two measurements. The test–retest reliability was stable. The structural validity was reasonable and effective. Additionally, convergent validity is satisfactory, while criterion validity is also satisfactory. The three-factor model consisting of depression, anxiety and stress was confirmed through CFA.

**Conclusion:**

The DASS-Y exhibits satisfactory psychometric properties among Chinese secondary school adolescents, reliably and appropriately screening for mental health issues such as depression and anxiety within this population. Consequently, it can be employed as a standard tool for routine mental health surveillance in secondary schools.

## Introduction

Mental health problems in adolescents have become one of the most important public health challenges of the 21st century (Williams et al., 2020). Globally, an estimated 10–20% of adolescents experience mental health conditions (Kieling et al., 2011; Feiss et al., 2019), with negative emotional disorders such as depression and anxiety being among the most prevalent psychiatric disorders (Patton et al., 2016). According to a meta-analysis on the mental health of children and adolescents in 27 countries and regions, the global prevalence of mental health issues in children and adolescents was 13.4%, with anxiety at 6.5%, depression at 2.6%, and major depression at 1.3% (Polanczyk et al., 2015). The prevalence of anxiety and depressive symptoms among Chinese children and adolescents is increasing due to rapid economic and social development (Cui et al., 2021). The latest data of 2022 show that the overall prevalence of mental health problems among Chinese school-age children and adolescents aged 6–16 is 17.5%, of which 4.7% are anxious, 3.0% are depressed, and 2.0% are severely depressed. Moreover, there is a high degree of comorbidity between depression and anxiety (Li et al., 2022). Early screening provides important opportunities for rapid identification and intervention for mental health problems such as depression and anxiety.

At present, many mental health measurement tools can be used to assess depression and anxiety symptoms in children and adolescents, such as the Patient Health Questionnaire-9 (PHQ-9) (Kroenke, 2021), the Child Anxiety and Depression Scale (CADS) (Loades et al., 2022), the Generalized Anxiety Scale-7 item (GAD-7) (Krause et al., 2021), the Beck Depression Inventory (BDI) (Smarr and Keefer, 2011), the Beck Anxiety Inventory (BAI) (Julian, 2011), However, while these measures can assess a mixture of emotional states and general distress symptoms, they have limited capacity to distinguish between depression and anxiety (Joiner et al., 1996). To compensate for these limitations, Lovibond P. F. and Lovibond (1995) and Lovibond S. H. and Lovibond (1995) developed the Depression Anxiety Stress Scales (DASS) and its short version DASS-21. Especially DASS-21, which was widely welcomed for its brevity, ease of completion and scoring (Antony et al., 1998). The DASS-21 is a simplified version of the DASS with a more streamlined test with 21 questions that screen for depression (7 questions), anxiety (7 questions), and stress (7 questions). Subjects are asked to use a 4-point combined severity/frequency scale to rate the extent to which they have experienced each item over the past week. The scale ranges from 0 (did not apply to me at all) to 3 (applied to me very much, or most of the time). Scores for Depression, Anxiety, and Stress are calculated by summing the scores for the relevant items and multiplying by two. The DASS-21 has been extensively validated and utilized in different cultures, domains, settings, and populations around the world due to its acceptable validity, reliability, simplicity, and convenience, internal consistency coefficient between 0.73 ~ 0.91 (Ronk et al., 2013; Vignola and Tucci, 2014; Park et al., 2020). DASS-21 has also been widely applied in China, showing excellent psychometrics in different populations across the country, and Cronbach’s a value ranged from 0.71 ~ 0.96 (Wang et al., 2016; Zou et al., 2018; Yu et al., 2023).

Since DASS-21 was originally developed with adults over the age of 18 years as the target population (Osman et al., 2012), it might not be appropriate to use it in adolescents under age of 18 years. Earlier studies have reported that the adult model DASS-21 cannot accurately distinguish the symptoms of depression and anxiety among adolescents in some cultural contexts (Shaw et al., 2017; Le et al., 2017). In addition, DASS-21 contains several expressions and words that may not be familiar to adolescents (Szabó, 2010), for example, questions such as “I felt that life was meaningless” and “I felt I wasn’t worth much as a person” may not be able to be accurately self-reported by Chinese adolescents, who are culturally introverted and subtle with an oriental cultural background. Szabo and Lovibond (2022) developed a screening tool based on DASS and DASS-21 for depression and anxiety symptoms specifically for children and adolescents under the age of 18: the Depression Anxiety Stress Scale for Youth (DASS-Y). The DASS-Y has comprising simplified wording, appropriate terminology, and has been validated and utilized in Australia (Mackenzie et al., 2023) and Serbian (Jovanović, 2024), showing good internal consistency (*a* = 0.86–0.94). However, these studies have shown that there exist differences in the factor structure and stability of the DASS-Y across ethnic populations.

The DASS-Y is specifically designed to measure emotional disorders such as depression and anxiety among adolescents, and assess a subject’s mental health status through specific scores, making it simpler and more practical than the current mental health measurement scales that test depression or anxiety singularly. However, there remains limited evidence regarding the application of DASS-Y among Chinese adolescent students in secondary schools, especially in terms of stability across time. Considering the differences in the expression of emotions, mental health concepts in Chinese and Western cultures and the expectations and pressures of Chinese society as well as the localization of the terminology of the survey items for processing and adaptation, the DASS-Y urgently needs to be validated in the Chinese adolescent population to confirm its applicability for screening depression and anxiety in the Chinese adolescent population. Therefore, the present study aimed to evaluate psychometric properties of DASS-Y in school adolescents in China.

## Methods

### Participants

A convenient random sampling method was used to select one secondary school from each of the status locations (urban and rural). Subsequently, within each selected school, one grade level was randomly chosen, and all students from the designated grade participated in the study.

We used the “Confidence Intervals for Coefficient Alpha” module of PASS 21 (NCSS, Ketchum) to determine the necessary sample size for the study. We estimate coefficient alpha with a two-sided 95% confidence interval with a width no wider than 0.1 and set examine values of *K* = 21 (scale items). From past studies, we want to use a planning estimate of 0.8 for the sample coefficient alpha. The necessary sample size for the study was determined to be 131. Considering the 10% rejection and withdrawal ratio, a total of 146 high school students were needed for this study.

No exclusion criteria were set and all students who were enrolled in the school and provided written informed consent from themselves and their parents/guardians were included in the study. Ultimately, we recruited a total of 326 students, including students from grade 9 in urban secondary schools and students from grade 10 in rural secondary schools, of which 163 (50%) were girls and 139 (42.6%) were in 9th grade. The mean age of the participants was 15.72 years (SD = 0.82, range: 14–18 years), the participating students in this study met the research design requirements.

### Measures

#### Baseline demographic characteristics

A self-administered questionnaire was used to collect basic demographic information such as gender, age (in years), grade and place of residence of the participants.

#### Depression Anxiety Stress Scale for Youth (DASS-Y)

The DASS-Y is a 21-tiems self-report measure containing three subscales of depression (7 items), anxiety (7 items), and stress (7 items). Subjects responded to each item by rating the frequency and severity of symptoms over the past week on a four-point Likert scale from 0 (not true) to 3 (very true). Each subscale was scored 0–21 and the total scale was scored 0–63. Higher scores indicate higher levels of symptoms in each domain and all items can also be added into a total score as a measure of general psychological distress. It is worth mentioning that, according to the use manual, the DASS-Y does not need to multiply the scores by 2 as per the DASS-21, because the DASS-Y is a standalone instrument with scores that cannot be directly compared to either of the adult questionnaires. Based on the scoring, the results are categorized into five levels: normal, mild, moderate, severe, and extremely severe (Supplementary Table S1).

Prior to this, as there is no Chinese version of the DASS-Y, the research group translated the DASS-Y into Chinese. First, the English version of the DASS-Y was translated into Chinese by two independent translators with backgrounds in studying and working in English-speaking countries, and then discussions were organized with mental health experts, epidemiologists, secondary school headmasters, class teachers and school counselors. Finally, the discussed version was translated back into English by the two researchers with bilingual backgrounds to check and reviewed by native English-speaking researchers to determine consistency with the original version.

The final Chinese version of DASS-Y was preliminarily tested among a small group of secondary school students (*n* = 10). No comprehension difficulties or ambiguities were found (data not shown in this study). The original DASS-Y can be obtained without charge from a website^1^, and the final Chinese version of the questionnaire is presented in the Appendix.

#### General Health Questionnaire-12 (GHQ-12)

The GHQ-12 is a self-report mental health status assessment tool. It evaluates the condition of the participant’s mental health in the past few weeks in terms of social relationships, physical pain, emotional state, sleep, anxiety, stress, and general feelings of well-being dimensions. The questionnaire has 12 items, of which 6 questions are positively scored and 6 questions are negatively scored, with 4 options, namely: “better than usual, same as usual, worse than usual, much worse than usual,” and scored from 0 to 3 according to the Likert 3-point scale, with a total of 0–36. A score greater than 3 indicates psychological distress, with higher scores indicating more serious mental health problems and can be classified as normal and abnormal based on the score (Shek, 1987). As the GHQ-12 contains self-ratings of the dimensions of general health experience, anxiety, and stress. The GHQ-12 has been widely validated and used (Guan and Han, 2019), with internal consistency Cronbach’s *a* of 0.721–0.903. We used the Zhang et al. (2008) supplemented GHQ-12, and its internal consistency Cronbach’s *a* in our test–retests was 0.853 and 0.872.

#### Pittsburgh Sleep Quality Index (PSQI)

The PSQI consists of a 19-item self-report scale that assesses sleep quality and sleep disorders in participants over the past few weeks. These items were used to calculate seven dimensions corresponding to specific sleep domains: subjective sleep quality, sleep latency, sleep duration, habitual sleep efficiency, sleep disorders, use of sleep aids and daytime dysfunction. Then, these dimensional scores were summed in order to generate a single PSQI score representing overall sleep quality, with a score of more than 5 indicating the presence of a sleep disorder, and classified into four levels of very good, good, poor, and very poor based on the scores (Buysse et al., 1989). The PSQI has been widely used and validated (Raniti et al., 2018) with internal consistency Cronbach’s *a* of 0.771–0.953. This study used the revised Chinese version of Liu et al. (1996), their internal consistency Cronbach’s *a* was 0.853 and 0.872 in our test–retest, respectively. As sleep has been shown to be highly correlated with emotional disorders such as depression and anxiety (Beaudequin et al., 2020), the criterion-related validity of DASS-Y was validated using the PSQI scale.

### Procedure

Data collection lasted for 2 months from March, 2022 to May, 2022. The ethical aspect was ensured by providing information to the adolescents and information sheet together with the consent form was sent to the family to obtain the consent of the family days before data collection.

Questionnaires were completed on school premises in a pen-and-paper written format. Following the distribution of the questionnaires, teachers read a standardized set of instructions asking the participants to read each item and select the most appropriate answer. Participants were reminded that the procedure was entirely voluntary, their responses were confidential, and there were no right or wrong answers. All students completed the same questionnaire for two times: enrollment (T_1_), and 5 weeks after enrollment (T_2_).

The study procedures were approved by the Ethics Committee of Fujian Provincial Hospital (K-2023-03-005).

### Statistical analysis

All statistical analyses were performed in SPSS (version 26, SPSS Inc., Chicago, IL) except the confirmatory factor analysis (CFA), which was conducted using AMOS (version 26). The internal consistency reliability of the DASS-Y was examined by calculating the McDonald’s omega (*ω*), Cronbach’s alpha, and intra-class correlation coefficient (ICC) with their 95% confidence intervals (95% CI) for the test–retest data. A split-half coefficient ranging from 0.70 to 0.80 indicates good internal consistency, between 0.60 and 0.70 is considered acceptable (Campbell, 1991). The ICC was calculated using the absolute agreement type of a two-way mixed model, with ICC > 0.75 indicating good reliability (Shrout and Fleiss, 1979). A Cronbach’s alpha >0.70 suggests good internal consistency and reliability, while >0.90 indicates very high internal consistency and excellent reliability (Olden et al., 2009).

The fit of the DASS-Y data was assessed through confirmatory factor analysis (CFA) using Maximum Likelihood (ML) estimation, in order to determine the appropriate fitting model for DASS-Y. The model’s fit was determined by various fit indices. Calculations included chi-square ($\chi^{2}$), Comparative Fit Index (CFI), Tucker-Lewis Index (TLI), and Root Mean Square Error of Approximation (RMSEA) with its 90% confidence interval (*CI*). Although $\chi^{2}$ was reported, they were not used as a criterion for model fit evaluation due to their sensitivity to smaller sample sizes (Bentler, 2007). The model fit was assessed using the following criteria: (1) Adequate fit: RMSEA <0.08, CFI and TLI > 0.90; (2) Good fit: RMSEA <0.05, CFI and TLI > 0.95 (Bentler, 1990). Furthermore, a final fitted model diagram of DASS-Y was plotted to further illustrate the standardized factor loadings among the various items of DASS-Y.

Finally, the convergent validity of DASS-Y was assessed by calculating the Average Variance Extracted (AVE) and Composite Reliability (CR) indices. Pearson correlation coefficients (*r*) were computed between DASS-Y, GHQ-12, and PSQI to evaluate the criterion validity of DASS-Y. AVE > 0.50 and CR > 0.70 indicate good validity, AVE > 0.4 and CR > 0.80 suggest fair validity of the scale (Fornell and Larcker, 1981).

## Results

### The factorial structure of the DASS-Y

For DASS-Y, the initial three-factor structural model was tested using CFA. As shown in Table 1, in the test–retest analysis, the CFI values were 0.903 and 0.890, the TLI values were 0.890 and 0.875, and the RMSEA values (90% *CI*) were 0.072 (0.064 ~ 0.080) and 0.078 (0.071 ~ 0.084), respectively. The fit indices show that the original DASS-Y three-factor model does not fully meet the criteria.

**Table 1:** DASS-Y original three-factor model fit indices.

Time	N	χ^2^	DF	CFI	TLI	RMSEA	RMSEA 90% *CI*
T_1_	326	499.004	186	0.903	0.890	0.072	0.064, 0.080
T_2_	326	513.782	186	0.890	0.875	0.078	0.071, 0.092

**Figure 1:**
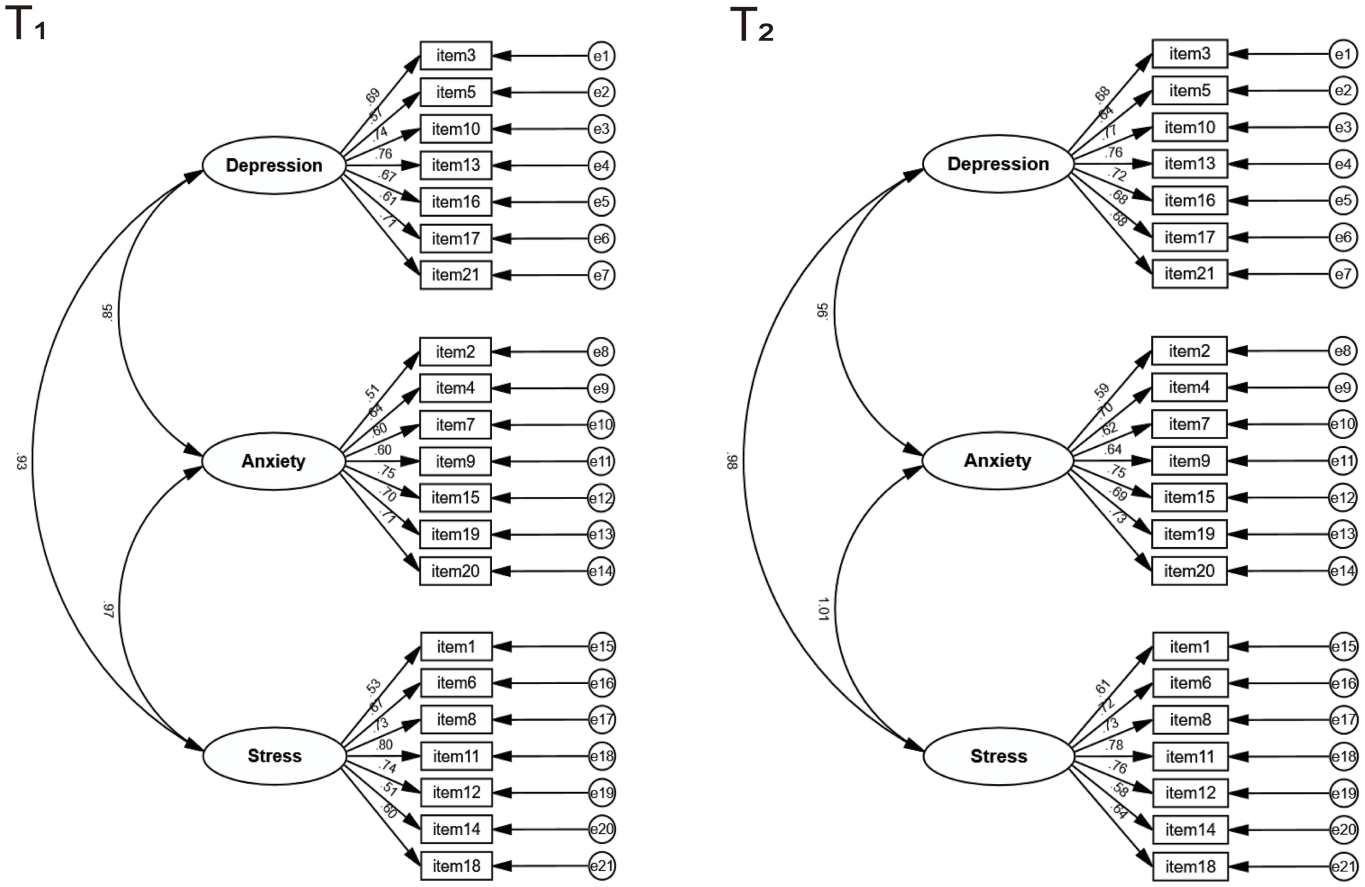
Path diagram of the 3-factor oblique DASS-Y model, with standardized parameter estimates. The lines indicate the three subscales of the DASS-Y depression, anxiety and stress and the factor structure and associations between the respective entries. The relationships between all pathways are presented as standardized pathway coefficients.

Similarly, as illustrated in Figure 1, the original three-factor oblique model depicts the factor loadings among the DASS-21 and its subscales. In the test–retest, items within each subscale exhibited satisfactory factor loadings (≥0.50), with robust factor loadings were observed between the three subscales (0.85 and 0.98), except for T_2_ for stress and anxiety. An anomalous value was observed between the Stress subscale and the Anxiety subscale in the T2 test (1.01).

### The internal consistency, test–retest reliability, and subscales-total correlation of the DASS-Y

As shown in Table 2, the McDonald’s omega (*ω*) of the DASS-Y scale across the test–retest were 0.935 and 0.948, respectively, with 0.853 and 0.871 for the depression subscale, 0.828 and 0.854 for the anxiety subscale, and 0.844 and 0.863 for the stress subscale. The Cronbach’s alpha was 0.935 and 0.948 for the total scale, with 0.853 and 0.871 for the depression subscale, 0.827 and 0.854 for the anxiety subscale, and 0.840 and 0.863 for the stress subscale. All subscales exhibited satisfactory domain-total correlations (*r* > 0.90). In addition to this, across the two measurements, the DASS-Y demonstrated good test–retest reliability, ICC = 0.901 (95% CI: 0.877–0.921), and the measurement results of each subscale were highly correlated (ICC > 0.85).

**Table 2:** Correlation coefficients for the reliability of DASS-Y and its subscales.

		*ω*	Cronbach’s *α*	Domain-total correlation	ICC (95% CI)
Depression	T_1_	0.853	0.853	0.910	0.897 (0.872 ~ 0.917)
	T_2_	0.871	0.871	0.928	
Anxiety	T_1_	0.828	0.827	0.923	0.872 (0.841 ~ 0.897)
	T_2_	0.854	0.854	0.948	
Stress	T_1_	0.844	0.840	0.943	0.855 (0.857 ~ 0.907)
	T_2_	0.863	0.863	0.950	
DASS-Y	T_1_	0.935	0.935		0.901 (0.877 ~ 0.921)
	T_2_	0.948	0.948		

### The convergent validity and criterion validity of the DASS-Y

Based on the Confirmatory Factor Analysis (CFA) model, the Average Variance Extracted (AVE) and Composite Reliability (CR) indices were calculated for each DASS-Y item on its corresponding subscale. In the test–retest, the AVE for each item on the depression subscale was 0.465 and 0.500, CR = 0.858 and 0.874, the AVE for each item on the anxiety subscale was 0.420 and 0.461, CR = 0.833 and 0.856, and the AVE for each item on the stress subscale was 0.439 and 0.476, CR = 0.842 and 0.864 (Supplementary Table S3).

As expected, the DASS-Y showed strong correlations with the GHQ-12, with Pearson correlation coefficients of 0.789 and 0.710 (*p* < 0.001) on test–retest, respectively. Depression (*r* = 0.721 and 0.694, *p* < 0.001), anxiety (*r* = 0.756 and 0.642, *p* < 0.001) and stress (*r* = 0.779 and 0.683, *p* < 0.001) were also strongly correlated with the GHQ-12. Meanwhile, the DASS-Y was also moderately correlated with the PSQI, the Pearson correlation coefficients of the DASS-Y and PSQI were 0.68 and 0.602 (*p* < 0.001) in both tests. Depression (*r* = 0.609 and 0.559, *p* < 0.001), anxiety (*r* = 0.578 and 0.575, *p* < 0.001) and stress (*r* = 0.639 and 0.577, *p* < 0.001) were also moderately correlated with the PSQI (Table 3).

**Table 3:** Correlations between DASS-Y and GHQ-12, and PSQI.

		Depression	Anxiety	Stress	DASS-Y
T_1_	GHQ-12	0.721^**^	0.756^**^	0.779^**^	0.789^**^
	PSQI	0.609^**^	0.578^**^	0.639^**^	0.668^**^
T_2_	GHQ-12	0.694^**^	0.642^**^	0.683^**^	0.710^**^
	PSQI	0.559^**^	0.575^**^	0.577^**^	0.602^**^

**p < 0.001.

## Discussion

Emotional disorders such as depression and anxiety are common mental health problems in adolescents. Early screening offers an important opportunity for prompt diagnosis and timely intervention. To the best of our knowledge, this is one of two studies of DASS-Y psychometric properties in Chinese adolescent populations.

Prior to this, only Cao et al. (2023) conducted a DASS-Y study in a population of Chinese children and adolescents. In contrast to their work, we examined the internal consistency reliability, test–retest reliability, factor structure, convergent validity, and criterion validity of DASS-Y by calculating McDonald’s Omega, Cronbach’s Alpha, and Intraclass Correlation Coefficient (ICC) between two measurements. These indicators align with the findings reported by Cao et al. (2023), suggested that the DASS-Y exhibited high internal consistency, stable test–retest reliability, and cross-temporal stability in the Chinese adolescent population.

We validated the factor structure and the original three-factor model of the DASS-Y. In terms of factor structure and factor loadings, the internal components of each subscale exhibit good factor loadings, similar to the findings of studies by Szabo and Lovibond (2022), Mackenzie et al. (2023), and Jovanović (2024), indicating that each dimension can adequately reflect symptoms of depression, anxiety, and stress. However, unlike them, there is an overlap of the stress subscales, which may be related to our cultural traditions, where stress is not a psychological problem but more of an emotional expression or, like anxiety, a worry about the future.

In terms of model fit, the original three-factor structural model did not fully conform to the established criteria, which is similar to previous findings (Cao et al., 2023). Hu and Bentler (1999) argue that all fit indices are intercept values assessed based on prior models, and their results may vary under different conditions, and that, in the real world, it is necessary to also take into account factors such as the sensitivity of the responses to the test items and small sample bias, among other factors. Therefore, this may be related to the source of our sample size, where students in different regions (urban and rural) may have inconsistent understanding of emotional disorders such as depression and anxiety, leading to bias in self-reported DASS-Y, which also affects the factor structure and construct validity of our DASS-Y. Considering that DASS-Y is a new scale with limited evidence in the Chinese population, we did not jump to conclusions after weighing the sample size (*n* ≤ 500), the study context, and the purpose of the study, and further validation is needed with larger and more diverse populations in the future.

In the analysis of the convergent validity of the DASS-Y, it was observed that only the AVE of the depression subscale of T_2_ reached a satisfactory level of 0.500. However, according to Fornell and Larcker (1981), CR greater than 0.6 is sufficient to establish convergent validity, even if the AVE falls below 0.5. Upon conducting tests and retests, it was found that the CR values of all subscales exceeded 0.8. Therefore, the research maintains that the DASS-Y scale possesses acceptable convergent validity and good combinatorial reliability in assessing mental health issues among Chinese adolescents. These findings further corroborate the robust convergent validity and stable test structure of the DASS-Y.

As shown in the results, the DASS-Y and the GHQ-12 exhibited strong correlations. Both instruments include core dimensions that measure negative emotional states, such as depression, anxiety, and stress, and assess mental health status through a total score. These instruments effectively identify the core symptoms of depression and anxiety, which are crucial for assessing the severity of mental health problem (Li et al., 2009). Additionally, the DASS-Y was found to have a moderate correlation with the PSQI. Previous research has demonstrated that negative emotions, including depression and anxiety, impact sleep and are strongly associated with sleep quality (Xue et al., 2022). These results confirm that the DASS-Y serves as an effective tool for screening mental health problem such as depression and anxiety among adolescents.

As a convenient, brief, and non-professionally administered instrument for assessing mental health problems, this study demonstrated that Chinese adolescent students could effectively comprehend the item content and verbal presentation of the DASS-Y. Furthermore, laypersons were able to organize and complete the questionnaire with greater ease during the testing process. Notably, the questionnaire’s completion time, averaging 10 min, fell within the acceptable range for the participating students. Consequently, the DASS-Y provides an alternative testing tool for screening mental health problems among Chinese children and adolescents, thereby enriching the assessment toolbox available for this population. These findings may suggest that the DASS-Y should be included as a regular part of the mental health examination in regular student health screening in schools to routinely monitor the mental health status of students.

### Limitations and future research

It is noteworthy that the present study also has some limitations. Firstly, the study specifically recruited students from grades 9 and 10, encompassing the junior high school and high school learning stages in China, with ages ranging from 14 to 18, however, this may limit the generalization of the findings to other age groups. Second, our study was conducted in only one region of China and lacked comparable data. Future studies could further enrich the dataset for cross-population, cross-region and cross-country comparisons. Thirdly, the DASS-Y is a self-report scale that reflects individual’s situation during the past week. Given that negative emotional problems vary over time and according to personal feelings, and that there is as yet universally established standard for the time interval between repeated measurements, multiple sources of reporting should be adopted in the future, including parent-reported scales, teacher-reported scales, and structured interviews. Fourth, sample geographic, cultural, and linguistic contexts affect factor constructs and modeling, and future DASS-Y studies should be conducted in different regions, populations, and cultural contexts to further validate the applicability of the DASS-Y in the Chinese adolescent population. Finally, we did not discuss the discriminant validity of DASS-Y, despite its significance, as it was not the primary objective of this study, however, further in-depth discussion should be pursued in future research.

## Conclusion

This study investigated the psychometric properties of the DASS-Y among Chinese adolescents. The results indicate that the DASS-Y demonstrates good internal consistency, test–retest stability, and correlational validity, has a reasonable factor structure, and can effectively differentiate depressive, anxiety, and stress symptoms among Chinese adolescents. These findings suggest that the DASS-Y can be a useful self-report measure for assessing common mental health problems (depression, anxiety) among Chinese secondary school students. We suggest that frontline teachers and school mental health counselors promote and utilize this valuable tool as an effective means of screening and assessing students’ mental health issues.

## Data Availability

The original contributions presented in the study are included in the article/Supplementary material, further inquiries can be directed to the corresponding author.

## Footnotes

^1^ https://dass.psy.unsw.edu.au/DASSY.htm
