# Supplementary material for: Validation and psychometric properties of the Depression Anxiety Stress Scale for Youth in Chinese adolescents
Source: Front Psychol. 2024 Nov 13;15:1466426. doi: 10.3389/fpsyg.2024.1466426 (PMC11602705; doi:10.3389/fpsyg.2024.1466426)
Supplement: Supplementary file 1 [file Data_Sheet_1.PDF]

Appendix

DASS-Y 量表

我们想了解您过去一周的感受。下面有一些问题，请您根据您过去一周的感受，在与您最相匹配的选项上打“√”。请注意，您的选择没有正确或错误之分。

问题	不符合（0）	有点符合（1）	比较符合（2）	非常符合（3）
1 我经常因为一些小事而苦恼				
2 我头晕眼花，浑身无力，感觉要晕倒了				
3 我对任何事情都提不起兴趣				
4 尽管没在运动，也没有生病，我也会呼吸困难（呼吸很快）				
5 我讨厌我的生活				
6 我对事情的反应很过度				
7 我感到手在发抖				
8 很多事情让我感到有压力				
9 我感到害怕				
10 我觉得没有什么值得期待的事情				
11 我很容易被激怒				
12 我很难放松下来				
13 我总是很悲观				

14 当我被别人打断时会很恼火

15 我觉得自己快要奔溃了

16 我恨我自己

17 我觉得自己一无是处

18 我很容易生气

19 即使没有做任何的剧烈运动，我

也觉得我的心跳很快

20 我感到莫名的恐惧

21 我觉得生活很糟糕
