# Supplementary material for: Validation and psychometric properties of the Depression Anxiety Stress Scale for Youth in Chinese adolescents
Source: Front Psychol. 2024 Nov 13;15:1466426. doi: 10.3389/fpsyg.2024.1466426 (PMC11602705; doi:10.3389/fpsyg.2024.1466426)
Supplement: Supplementary file 2 [file Table_1.DOCX]

**Supplemental Table 1**

Cutoff scores for the DASS-Y scales and total scores.

|  | **Depression** | **Anxiety** | **Stress** | **Total** |
| --- | --- | --- | --- | --- |
| Normal | 0-6 | 0-5 | 0-11 | 0-23 |
| Mild | 7-8 | 6-7 | 12-13 | 24-29 |
| Moderate | 9-13 | 8-12 | 14-16 | 30-39 |
| Severe | 14-16 | 13-15 | 17-18 | 40-46 |
| Extremely severe | 17+ | 16+ | 19+ | 47+ |

*Note：The DASS-Y is divided into three subscales of depression, anxiety and stress and is graded by scores on five scales: normal, mild, moderate, severe and extremely severe.*

**Supplemental Table 2**

Convergence validity indices for each subscale of DASS-Y (CFA model).

| **Path relationship** | | |  | **T_1_** | | |  | **T_2_** | | |
| --- | --- | --- | --- | --- | --- | --- | --- | --- | --- | --- |
|  |  |  |  | **Estimate** | **AVE** | **CR** |  | **Estimate** | **AVE** | **CR** |
| item3 | <--- | Depression |  | 0.692 | 0.465 | 0.858 |  | 0.683 | 0.500 | 0.874 |
| item5 | <--- |  |  | 0.569 |  |  |  | 0.639 |  |  |
| item10 | <--- |  |  | 0.736 |  |  |  | 0.771 |  |  |
| item13 | <--- |  |  | 0.763 |  |  |  | 0.763 |  |  |
| item16 | <--- |  |  | 0.670 |  |  |  | 0.724 |  |  |
| item17 | <--- |  |  | 0.614 |  |  |  | 0.682 |  |  |
| item21 | <--- |  |  | 0.711 |  |  |  | 0.676 |  |  |
|  | | | | | | | | | | |
| item2 | <--- | Anxiety |  | 0.514 | 0.420 | 0.833 |  | 0.588 | 0.461 | 0.856 |
| item4 | <--- |  |  | 0.637 |  |  |  | 0.702 |  |  |
| item7 | <--- |  |  | 0.596 |  |  |  | 0.622 |  |  |
| item9 | <--- |  |  | 0.600 |  |  |  | 0.642 |  |  |
| item15 | <--- |  |  | 0.749 |  |  |  | 0.753 |  |  |
| item19 | <--- |  |  | 0.696 |  |  |  | 0.692 |  |  |
| item20 | <--- |  |  | 0.715 |  |  |  | 0.735 |  |  |
|  | | | | | | | | | | |
| item1 | <--- | Stress |  | 0.535 | 0.439 | 0.842 |  | 0.607 | 0.476 | 0.864 |
| item6 | <--- |  |  | 0.667 |  |  |  | 0.720 |  |  |
| item8 | <--- |  |  | 0.729 |  |  |  | 0.728 |  |  |
| item11 | <--- |  |  | 0.801 |  |  |  | 0.777 |  |  |
| item12 | <--- |  |  | 0.743 |  |  |  | 0.759 |  |  |
| item14 | <--- |  |  | 0.511 |  |  |  | 0.577 |  |  |
| item18 | <--- |  |  | 0.596 |  |  |  | 0.643 |  |  |

***Note:*** *CFA= Confirmatory Factor Analysis, AVE= Average Variance Extracted, CR= Composite Reliability.*
